# 2-[(3,3-Di­methyl­indolin-2-yl­idene)meth­yl]-4-[(3,3-dimethyl-3*H*-indol-1-ium-2-yl)methyl­idene]-3-oxo­cyclo­but-1-en-1-olate chloro­form disolvate

**DOI:** 10.1107/S1600536813010386

**Published:** 2013-04-27

**Authors:** Graham Smith, Daniel E. Lynch

**Affiliations:** a Science and Engineering Faculty, Queensland University of Technology, GPO Box 2434, Brisbane, Queensland 4001, Australia; b Exilica Limited, The Technocentre, Puma Way, Coventry CV1 2TT, England

## Abstract

In the title squaraine dye solvate, C_26_H_24_N_2_O_2_·2CHCl_3_, the dye mol­ecule is essentially planar, except for the methyl groups, having a maximum deviation over the 26-membered delocalized bond system of 0.060 (2) Å. It possesses crystallographic twofold rotational symmetry with the indole ring systems adopting a *syn* conformation. The mol­ecular structure features intra­molecular N—H⋯O hydrogen bonds enclosing conjoint *S*7 ring motifs about one of the dioxo­cyclo­butene O atoms, while the two chloro­form solvent mol­ecules are linked to the second O atom through C—H⋯O hydrogen bonds.

## Related literature

For the first report of bis­(indole­nine)squaraine dyes with alkyl substituents on the *N*-atom of each of the indole­nine rings, see: Sprenger Von & Ziegenbein (1967 ▶). For background to bis­(indole­nine)squaraine dyes as biomarkers, see: Patsenker *et al.* (2011 ▶); Sameiro & Gonçalves (2009 ▶). For the structures of some analogues of the parent dye, see: Kobiyashi *et al.* (1986 ▶); Natsukawa & Nakazumi (1993 ▶); Tong & Peng (1999 ▶); Lynch & Byriel (1999 ▶); Lynch (2002 ▶); Arunkumar *et al.* (2007 ▶); Matsui *et al.* (2012 ▶); Lynch *et al.* (2012 ▶).
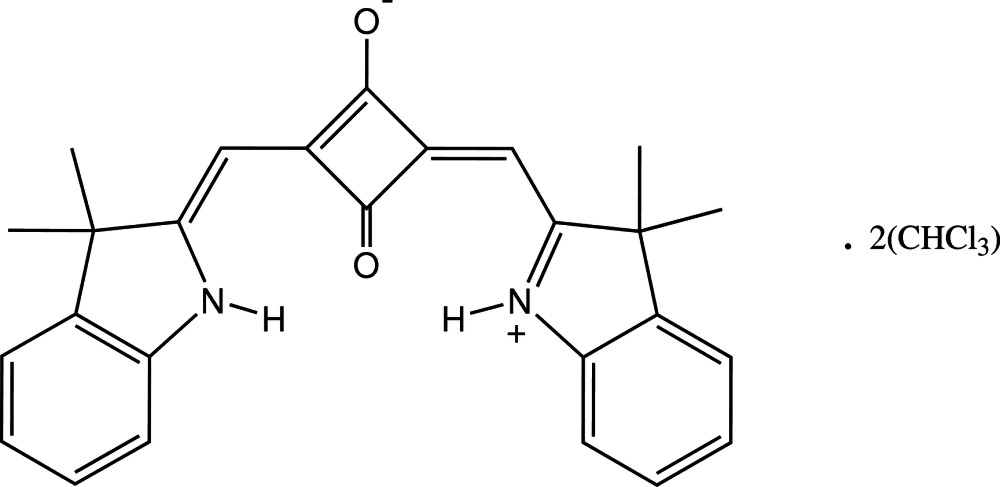


## Experimental

### 

#### Crystal data

C_26_H_24_N_2_O_2_·2CHCl_3_
*M* *_r_* = 635.21
Monoclinic, 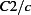

*a* = 20.4270 (11) Å
*b* = 13.5433 (5) Å
*c* = 11.4259 (5) Å
β = 109.561 (5)°
*V* = 2978.5 (3) Å^3^
*Z* = 4
Mo *K*α radiation
μ = 0.61 mm^−1^
*T* = 200 K
0.40 × 0.22 × 0.20 mm

#### Data collection

Oxford Diffraction Gemini-S CCD-detector diffractometer
Absorption correction: multi-scan (*CrysAlis PRO*; Agilent, 2012 ▶) *T*_min_ = 0.794, *T*_max_ = 0.888
10054 measured reflections
2926 independent reflections
2415 reflections with *I* > 2σ(*I*)
*R*_int_ = 0.027

#### Refinement

*R*[*F* ^2^ > 2σ(*F* ^2^)] = 0.044
*wR*(*F* ^2^) = 0.112
*S* = 1.02
2926 reflections
176 parameters
H-atom parameters constrained
Δρ_max_ = 0.54 e Å^−3^
Δρ_min_ = −0.50 e Å^−3^

### 

Data collection: *CrysAlis PRO* (Agilent, 2012 ▶); cell refinement: *CrysAlis PRO*; data reduction: *CrysAlis PRO*; program(s) used to solve structure: *SHELXS97* (Sheldrick, 2008 ▶); program(s) used to refine structure: *SHELXL97* (Sheldrick, 2008 ▶); molecular graphics: *PLATON* (Spek, 2009 ▶); software used to prepare material for publication: *PLATON*.

## Supplementary Material

Crystal structure: contains datablock(s) global, I. DOI: 10.1107/S1600536813010386/su2588sup1.cif

Structure factors: contains datablock(s) I. DOI: 10.1107/S1600536813010386/su2588Isup2.hkl

Supplementary material file. DOI: 10.1107/S1600536813010386/su2588Isup3.cml

Additional supplementary materials:  crystallographic information; 3D view; checkCIF report

## Figures and Tables

**Table 1:** Hydrogen-bond geometry (Å, °)

*D*—H⋯*A*	*D*—H	H⋯*A*	*D*⋯*A*	*D*—H⋯*A*
N1—H1⋯O2	0.88	1.96	2.7835 (18)	156
C15—H15⋯O1	0.98	2.13	3.075 (3)	161

**Fig. 1.**
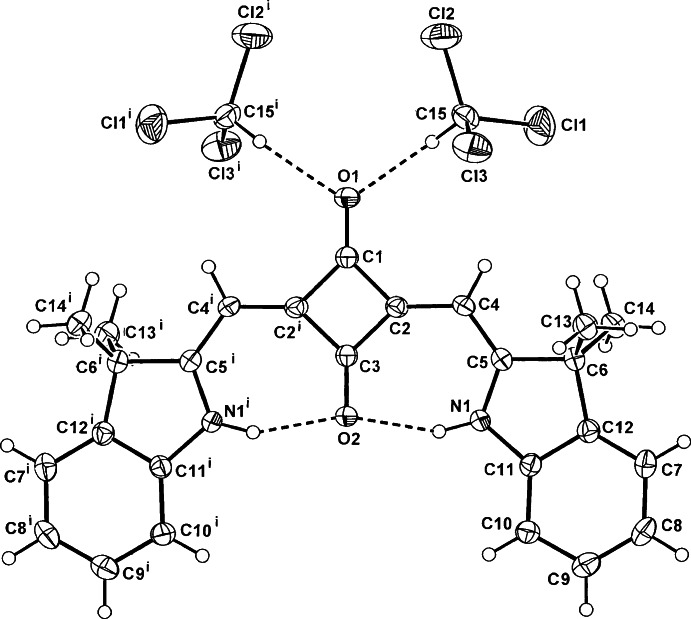
The molecular conformation and atom-numbering scheme for the title compound (symmetry code: (i) -x, y, -z + 3/2). The displacement ellipsoids are drawn at the 40% probability level. The intra- and inter-species N-H···O and C-H···O hydrogen bonds are shown as dashed lines.

## supplementary crystallographic information

### Comment

Bis(indolenine)squaraine dyes, in which there is an alkyl substituent on the *N*-atom of each of the indolenine rings, were first reported on by Sprenger Von & Ziegenbein (1967) and have been studied since then for a range of opto-electronic applications such as long-wavelength protein-sensitive bioprobes (Lynch *et al.*, 2012; Patsenker *et al.*, 2011; Sameiro & Gonçalves, 2009). However, the parent dye compound 2,4-[(3,3-dimethyl-2-indolylidene)methyl]cyclobutenediylio-1,3-diolate, which has no *N*-alkyl substituent (*R*) on the indolenine ring, has remained relatively untouched in the literature, including reporting of the crystal structure. The crystal structures of a number of analogues with such substituents have been reported; for *e.g. R* = methyl (Kobiyashi *et al.*, 1986), ethyl (Natsukawa & Nakazumi, 1993), isopropyl (Tong & Peng, 1999), *n*-butyl (Matsui *et al.*, 2012), *n*-hexyl (Lynch & Byriel, 1999) and *n*-octyl (Lynch, 2002).

Evaporation of a solution of the dye in chloroform gave the title compound solvate as large green-black crystal prisms and its crystal structure is reported on herein. The dye molecule adopts the uncommon *syn*-conformation with respect to the indolenine rings, having crystallographic twofold rotational symmetry (Fig. 1). The structures of all other members of this series of *N*-alkyl-substituted squaraine dyes have the inversion-related *anti*-conformation.

The planarity of the delocalized 26-membered linked ring system in the overall molecule is indicated by maximum deviations of 0.059 (2) (C6 and C6^i^) and 0.060 (2) (C4 and C4^i^) from the least-squares plane [symmetry code (i): -*x*, *y*, -*z* + 3/2]. This planarity is further stabilized by the intramolecular N—H···O hydrogen bonds to O2 of the dioxocyclobutene ring (Table 1), closing conjoint *S*7 ring motifs.

Inter-species C—H···O hydrogen-bonding interactions link the two chloroform molecules to the second O-atom (O1). Although chloroform is a common solvent for the crystallization of squaraine dyes, chloroform solvates are uncommon with only three such structures reported on previously (Natsukawa & Nakazumi, 1993; Lynch & Byriel, 1999; Arunkumar *et al.*, 2007).

### Experimental

Squaric acid (200 mg, 1.75 mmol) was added to 2.0 molar equivalents of 2,3,3-trimethylindolenine (0.56 g, 3.5 mmol) and quinoline (0.45 g, 3.5 mmol) in a 1:1 *v*/*v* mix of 1-butanol:toluene (30 ml) and the mixture was then refluxed for 16 h using a Dean and Stark apparatus. Upon cooling, metallic green crystals were collected *in vacuo*, washed with petroleum ether (60/40), and were used without further purification [Yield: 0.31 g (45%)]. Spectroscopic data are available in the archived CIF. For the X-ray diffraction analysis large green-black lustrous crystal prisms of the title compound were obtained from the room temperature evaporation of a solution of the dye in chloroform. A cleaved crystal specimen was used for the actual analysis.

### Refinement

The H atom of the N—H group was located in a difference Fourier but was subsequently refined as a riding atom: N-H = 0.88 Å with *U*_iso_(H) = 1.2*U*_eq_(N). C-bound H atoms were included in calculated positions and refined as riding atoms: C—H = 0.93 Å (aromatic or ethylenic), 0.96 Å (methyl) or 0.98 Å (methine) with *U*_iso_(H) = 1.5*U*_eq_(C-methyl) and = 1.2*U*_eq_(C) for other H atoms.

### Crystal data

**Table:**

C_26_H_24_N_2_O_2_·2CHCl_3_	*F*(000) = 1304
*M**_r_* = 635.21	*D*_x_ = 1.416 Mg m^−^^3^
Monoclinic, *C*2/*c*	Mo *K*α radiation, λ = 0.71073 Å
Hall symbol: -C 2yc	Cell parameters from 2690 reflections
*a* = 20.4270 (11) Å	θ = 3.3–28.8°
*b* = 13.5433 (5) Å	µ = 0.61 mm^−^^1^
*c* = 11.4259 (5) Å	*T* = 200 K
β = 109.561 (5)°	Prism, green
*V* = 2978.5 (3) Å^3^	0.40 × 0.22 × 0.20 mm
*Z* = 4	

### Data collection

**Table:**

Oxford Diffraction Gemini-S CCD-detector diffractometer	2926 independent reflections
Radiation source: Enhance (Mo) X-ray source	2415 reflections with *I* > 2σ(*I*)
Graphite monochromator	*R*_int_ = 0.027
Detector resolution: 16.077 pixels mm^-1^	θ_max_ = 26.0°, θ_min_ = 3.5°
ω scans	*h* = −22→25
Absorption correction: multi-scan (*CrysAlis PRO*; Agilent, 2012)	*k* = −16→16
*T*_min_ = 0.794, *T*_max_ = 0.888	*l* = −13→14
10054 measured reflections	

### Refinement

**Table:**

Refinement on *F*^2^	Primary atom site location: structure-invariant direct methods
Least-squares matrix: full	Secondary atom site location: difference Fourier map
*R*[*F*^2^ > 2σ(*F*^2^)] = 0.044	Hydrogen site location: inferred from neighbouring sites
*wR*(*F*^2^) = 0.112	H-atom parameters constrained
*S* = 1.02	*w* = 1/[σ^2^(*F*_o_^2^) + (0.047*P*)^2^ + 4.0361*P*] where *P* = (*F*_o_^2^ + 2*F*_c_^2^)/3
2926 reflections	(Δ/σ)_max_ < 0.001
176 parameters	Δρ_max_ = 0.54 e Å^−^^3^
0 restraints	Δρ_min_ = −0.50 e Å^−^^3^

### Special details

**Table:**

Experimental. Spectroscopic details of the as synthesized metallic green crystals of the title dye: UV/Vis (CHCl_3_), recorded on a Nicolet 205 F T—IR spectrometer: λmax (log ε): 665(5.54). IR (KBr, cm^-1^) recorded on a Unicam UV-4 spectrometer: λmax: 1623 (C—O). Electrospray mass spectra recorded in the the positive (ES+) ion mode: 397.1 [M+H]^+^, 460.1 [M+Na+MeCN]^+^, 815.3 [2M+Na]^+^, 1211.6 [3M+Na]^+^.
Geometry. Bond distances, angles *etc*. have been calculated using the rounded fractional coordinates. All su's are estimated from the variances of the (full) variance-covariance matrix. The cell e.s.d.'s are taken into account in the estimation of distances, angles and torsion angles
Refinement. Refinement of *F*^2^ against ALL reflections. The weighted *R*-factor *wR* and goodness of fit *S* are based on *F*^2^, conventional *R*-factors *R* are based on *F*, with *F* set to zero for negative *F*^2^. The threshold expression of *F*^2^ > σ(*F*^2^) is used only for calculating *R*-factors(gt) *etc*. and is not relevant to the choice of reflections for refinement. *R*-factors based on *F*^2^ are statistically about twice as large as those based on *F*, and *R*- factors based on ALL data will be even larger.

### Fractional atomic coordinates and isotropic or equivalent isotropic displacement parameters (Å^2^)

**Table:**

	*x*	*y*	*z*	*U*_iso_*/*U*_eq_	
O1	0.00000	0.35895 (15)	0.75000	0.0420 (8)	
O2	0.00000	0.02418 (14)	0.75000	0.0330 (7)	
N1	0.08460 (9)	0.01681 (12)	0.60326 (15)	0.0292 (5)	
C1	0.00000	0.2681 (2)	0.75000	0.0308 (9)	
C2	0.03058 (10)	0.19196 (14)	0.69306 (18)	0.0283 (6)	
C3	0.00000	0.1172 (2)	0.75000	0.0273 (8)	
C4	0.07197 (11)	0.19354 (15)	0.61835 (19)	0.0300 (6)	
C5	0.09788 (10)	0.11037 (14)	0.57941 (18)	0.0272 (6)	
C6	0.14808 (11)	0.10979 (15)	0.50618 (19)	0.0298 (6)	
C7	0.19200 (12)	−0.05112 (17)	0.4256 (2)	0.0364 (7)	
C8	0.18945 (12)	−0.15368 (18)	0.4251 (2)	0.0400 (8)	
C9	0.15195 (12)	−0.20386 (17)	0.4872 (2)	0.0390 (7)	
C10	0.11502 (12)	−0.15347 (15)	0.55071 (19)	0.0333 (7)	
C11	0.11792 (11)	−0.05144 (15)	0.54957 (18)	0.0278 (6)	
C12	0.15568 (10)	−0.00001 (15)	0.48861 (18)	0.0293 (6)	
C13	0.21729 (12)	0.15545 (17)	0.5872 (2)	0.0397 (7)	
C14	0.11833 (13)	0.16552 (17)	0.3829 (2)	0.0400 (8)	
Cl1	0.10014 (5)	0.46856 (7)	0.50703 (7)	0.0756 (3)	
Cl2	0.09549 (5)	0.60290 (5)	0.70002 (8)	0.0724 (3)	
Cl3	0.17949 (4)	0.42725 (6)	0.76203 (7)	0.0650 (3)	
C15	0.10219 (14)	0.47904 (18)	0.6610 (2)	0.0444 (8)	
H1	0.05820	0.00030	0.64720	0.0350*	
H4	0.08300	0.25470	0.59290	0.0360*	
H7	0.21760	−0.01770	0.38450	0.0440*	
H8	0.21330	−0.18910	0.38230	0.0480*	
H9	0.15150	−0.27250	0.48640	0.0470*	
H10	0.08950	−0.18680	0.59210	0.0400*	
H131	0.23420	0.12070	0.66480	0.0600*	
H132	0.21030	0.22370	0.60240	0.0600*	
H133	0.25060	0.15050	0.54490	0.0600*	
H141	0.15020	0.16100	0.33750	0.0600*	
H142	0.11160	0.23360	0.39920	0.0600*	
H143	0.07460	0.13680	0.33480	0.0600*	
H15	0.06270	0.44250	0.66990	0.0530*	

### Atomic displacement parameters (Å^2^)

**Table:**

	*U*^11^	*U*^22^	*U*^33^	*U*^12^	*U*^13^	*U*^23^
O1	0.0502 (14)	0.0225 (10)	0.0651 (15)	0.0000	0.0351 (12)	0.0000
O2	0.0396 (12)	0.0231 (10)	0.0439 (12)	0.0000	0.0240 (10)	0.0000
N1	0.0351 (9)	0.0247 (9)	0.0344 (9)	0.0005 (7)	0.0203 (8)	0.0024 (7)
C1	0.0302 (15)	0.0265 (15)	0.0392 (16)	0.0000	0.0161 (13)	0.0000
C2	0.0261 (10)	0.0252 (10)	0.0342 (11)	−0.0004 (8)	0.0111 (9)	0.0009 (8)
C3	0.0260 (14)	0.0265 (15)	0.0304 (14)	0.0000	0.0108 (12)	0.0000
C4	0.0336 (11)	0.0229 (10)	0.0377 (11)	−0.0023 (8)	0.0176 (9)	0.0028 (8)
C5	0.0268 (10)	0.0281 (10)	0.0272 (10)	−0.0018 (8)	0.0099 (8)	0.0017 (8)
C6	0.0315 (11)	0.0308 (11)	0.0310 (10)	−0.0012 (9)	0.0156 (9)	0.0018 (8)
C7	0.0336 (12)	0.0442 (13)	0.0349 (11)	0.0034 (10)	0.0162 (10)	0.0003 (9)
C8	0.0399 (13)	0.0444 (14)	0.0373 (12)	0.0119 (11)	0.0150 (10)	−0.0059 (10)
C9	0.0432 (13)	0.0337 (12)	0.0375 (12)	0.0079 (10)	0.0102 (10)	−0.0026 (9)
C10	0.0392 (12)	0.0288 (11)	0.0325 (11)	−0.0001 (9)	0.0127 (9)	0.0009 (8)
C11	0.0291 (10)	0.0295 (10)	0.0254 (9)	0.0031 (8)	0.0098 (8)	0.0002 (8)
C12	0.0272 (10)	0.0315 (11)	0.0303 (10)	0.0004 (9)	0.0110 (8)	0.0009 (8)
C13	0.0350 (12)	0.0408 (13)	0.0477 (13)	−0.0062 (10)	0.0196 (11)	−0.0029 (10)
C14	0.0524 (15)	0.0354 (12)	0.0366 (12)	0.0007 (11)	0.0206 (11)	0.0076 (9)
Cl1	0.0984 (7)	0.0894 (6)	0.0439 (4)	−0.0101 (5)	0.0303 (4)	−0.0026 (4)
Cl2	0.0981 (6)	0.0384 (4)	0.0764 (5)	−0.0049 (4)	0.0234 (5)	−0.0064 (3)
Cl3	0.0658 (5)	0.0531 (4)	0.0653 (5)	−0.0084 (4)	0.0077 (4)	0.0035 (3)
C15	0.0548 (15)	0.0384 (13)	0.0436 (13)	−0.0135 (12)	0.0214 (12)	−0.0011 (10)

### Geometric parameters (Å, º)

**Table:**

Cl1—C15	1.751 (2)	C7—C8	1.390 (3)
Cl2—C15	1.753 (3)	C8—C9	1.384 (3)
Cl3—C15	1.760 (3)	C9—C10	1.389 (3)
O1—C1	1.230 (3)	C10—C11	1.383 (3)
O2—C3	1.260 (3)	C11—C12	1.387 (3)
N1—C5	1.343 (3)	C4—H4	0.9300
N1—C11	1.405 (3)	C7—H7	0.9300
N1—H1	0.8800	C8—H8	0.9300
C1—C2^i^	1.466 (3)	C9—H9	0.9300
C1—C2	1.466 (3)	C10—H10	0.9300
C2—C4	1.388 (3)	C13—H133	0.9600
C2—C3	1.453 (3)	C13—H131	0.9600
C4—C5	1.380 (3)	C13—H132	0.9600
C5—C6	1.525 (3)	C14—H141	0.9600
C6—C12	1.516 (3)	C14—H142	0.9600
C6—C13	1.537 (3)	C14—H143	0.9600
C6—C14	1.533 (3)	C15—H15	0.9800
C7—C12	1.380 (3)		
			
C5—N1—C11	111.84 (18)	C6—C12—C7	131.1 (2)
C11—N1—H1	124.00	C6—C12—C11	109.10 (18)
C5—N1—H1	124.00	C2—C4—H4	118.00
C2—C1—C2^i^	90.59 (19)	C5—C4—H4	118.00
O1—C1—C2^i^	134.70 (11)	C8—C7—H7	121.00
O1—C1—C2	134.70 (11)	C12—C7—H7	121.00
C1—C2—C3	88.88 (16)	C7—C8—H8	119.00
C1—C2—C4	134.41 (18)	C9—C8—H8	120.00
C3—C2—C4	136.70 (18)	C8—C9—H9	119.00
O2—C3—C2	134.18 (11)	C10—C9—H9	119.00
C2—C3—C2^i^	91.64 (19)	C9—C10—H10	122.00
O2—C3—C2^i^	134.18 (11)	C11—C10—H10	122.00
C2—C4—C5	124.33 (19)	C6—C13—H131	109.00
N1—C5—C4	125.4 (2)	C6—C13—H132	110.00
N1—C5—C6	108.97 (17)	C6—C13—H133	109.00
C4—C5—C6	125.59 (18)	H131—C13—H132	110.00
C5—C6—C12	101.26 (16)	H131—C13—H133	110.00
C12—C6—C13	111.12 (18)	H132—C13—H133	109.00
C12—C6—C14	112.84 (17)	C6—C14—H141	109.00
C13—C6—C14	110.92 (18)	C6—C14—H142	109.00
C5—C6—C13	108.62 (17)	C6—C14—H143	109.00
C5—C6—C14	111.66 (19)	H141—C14—H142	109.00
C8—C7—C12	118.5 (2)	H141—C14—H143	109.00
C7—C8—C9	121.0 (2)	H142—C14—H143	110.00
C8—C9—C10	121.2 (2)	Cl1—C15—Cl2	110.82 (13)
C9—C10—C11	117.0 (2)	Cl1—C15—Cl3	109.94 (16)
N1—C11—C12	108.72 (17)	Cl2—C15—Cl3	110.12 (13)
C10—C11—C12	122.6 (2)	Cl1—C15—H15	109.00
N1—C11—C10	128.7 (2)	Cl2—C15—H15	109.00
C7—C12—C11	119.8 (2)	Cl3—C15—H15	109.00
			
C11—N1—C5—C4	179.2 (2)	C4—C5—C6—C12	−178.6 (2)
C11—N1—C5—C6	−2.7 (2)	C4—C5—C6—C13	64.3 (3)
C5—N1—C11—C10	−177.9 (2)	C4—C5—C6—C14	−58.3 (3)
C5—N1—C11—C12	0.8 (2)	C5—C6—C12—C7	177.2 (2)
O1—C1—C2—C3	180.00 (2)	C5—C6—C12—C11	−2.8 (2)
O1—C1—C2—C4	0.9 (3)	C13—C6—C12—C7	−67.6 (3)
C2^i^—C1—C2—C3	0.00 (12)	C13—C6—C12—C11	112.4 (2)
C2^i^—C1—C2—C4	−179.1 (2)	C14—C6—C12—C7	57.7 (3)
C2—C1—C2^i^—C3	0.02 (16)	C14—C6—C12—C11	−122.3 (2)
C1—C2—C3—O2	180.00 (1)	C12—C7—C8—C9	−0.6 (3)
C1—C2—C3—C2^i^	0.00 (11)	C8—C7—C12—C6	−179.9 (2)
C4—C2—C3—O2	−0.9 (3)	C8—C7—C12—C11	0.1 (3)
C4—C2—C3—C2^i^	179.1 (3)	C7—C8—C9—C10	0.8 (4)
C1—C2—C4—C5	176.79 (19)	C8—C9—C10—C11	−0.4 (3)
C3—C2—C4—C5	−1.9 (4)	C9—C10—C11—N1	178.4 (2)
C2—C4—C5—N1	3.0 (3)	C9—C10—C11—C12	−0.1 (3)
C2—C4—C5—C6	−174.8 (2)	N1—C11—C12—C6	1.5 (2)
N1—C5—C6—C12	3.3 (2)	N1—C11—C12—C7	−178.52 (19)
N1—C5—C6—C13	−113.77 (19)	C10—C11—C12—C6	−179.7 (2)
N1—C5—C6—C14	123.59 (19)	C10—C11—C12—C7	0.2 (3)

Symmetry code: (i) −*x*, *y*, −*z*+3/2.

### Hydrogen-bond geometry (Å, º)

**Table:**

*D*—H···*A*	*D*—H	H···*A*	*D*···*A*	*D*—H···*A*
N1—H1···O2	0.88	1.96	2.7835 (18)	156
C15—H15···O1	0.98	2.13	3.075 (3)	161

## References

[bb1] Agilent (2012). *CrysAlis PRO* Agilent Technologies Ltd, Yarnton, England.

[bb2] Arunkumar, E., Sudeep, P. K., Kamat, P. V., Noll, B. C. & Smith, B. D. (2007). *New J. Chem.* **31**, 677–683. 10.1039/b616224j PMC2849118 20376333

[bb3] Kobiyashi, Y., Goto, M. & Kurahashi, M. (1986). *Bull. Chem. Soc. Jpn*, **59**, 311–312.

[bb4] Lynch, D. E. (2002). *Acta Cryst.* E**58**, o1025–o1027.

[bb5] Lynch, D. E. & Byriel, K. A. (1999). *Cryst. Eng.* **2**, 225–239.

[bb6] Lynch, D. E., Kirkham, V. B., Chowdhury, M. Z. H., Wane, E. S. & Heptinstall, J. (2012). *Dyes Pigments*, **60**, 393–402.

[bb7] Matsui, M., Fukushima, M., Kubota, Y., Funabiki, K. & Shiro, M. (2012). *Tetrahedron*, **68**, 1931–1935.

[bb8] Natsukawa, K. & Nakazumi, H. (1993). *Sangyo Gij. Sogo Kenk. Hokuku*, **6**, 16–21.

[bb9] Patsenker, L. D., Tatarets, A. L., Povrozin, Y. A. & Terpetschnig, E. A. (2011). *Bioanal. Rev.* **3**, 115–137.

[bb10] Sameiro, M. & Gonçalves, T. (2009). *Chem. Rev.* **109**, 190–212. 10.1021/cr0783840 19105748

[bb11] Sheldrick, G. M. (2008). *Acta Cryst.* A**64**, 112–122. 10.1107/S0108767307043930 18156677

[bb12] Spek, A. L. (2009). *Acta Cryst.* D**65**, 148–155. 10.1107/S090744490804362X PMC2631630 19171970

[bb13] Sprenger Von, H.-E. & Ziegenbein, W. (1967). *Angew. Chem.* **79**, 581–582.

[bb14] Tong, L. & Peng, B.-X. (1999). *Dyes Pigments*, **43**, 73–76.

